# Atherogenic lipid indices and diabetic retinopathy in type 2 diabetes: a systematic review and meta-analysis

**DOI:** 10.3389/fmed.2025.1699408

**Published:** 2026-01-09

**Authors:** Ye Liang, Min Zuo, Chaoyang Wang, Lei Zhu

**Affiliations:** General Medical 3201 Hospital, Hanzhong, Shaanxi, China

**Keywords:** atherogenic index of plasma, diabetic retinopathy, meta-analysis, TG/HDL-C ratio, type 2 diabetes

## Abstract

**Objective:**

This study conducts a quantitative synthesis of existing evidence to evaluate the association of two novel atherogenic lipid indices—the triglyceride to high-density lipoprotein cholesterol ratio (TG/HDL-C) and the atherogenic index of plasma (AIP)—with the prevalence and severity of diabetic retinopathy (DR) in individuals with type 2 diabetes mellitus (T2DM).

**Methods:**

We systematically searched Cochrane Library, PubMed, Web of Science, Embase, CBM, CNKI, Wanfang, and Weipu for observational studies published before July 30, 2025. Cross-sectional, cohort, and case–control studies reporting AIP or TG/HDL-C data in T2DM patients with or without DR were eligible. Given the anticipated clinical and methodological diversity across observational studies, we prespecified a random-effects meta-analysis as the primary model, estimating τ^2^ via restricted maximum likelihood and constructing confidence intervals using the Hartung–Knapp–Sidik–Jonkman adjustment. Between-study variance (τ^2^) and 95% prediction intervals (PIs) were reported under the random-effects framework when at least three studies contributed to a given contrast, and fixed-effect models were additionally fitted as sensitivity analyses alongside heterogeneity, sensitivity, and publication bias assessments.

**Results:**

A total of 10 studies (*n* = 5,071) were included. In the unadjusted pooled analysis, individuals with DR had higher AIP values than those without DR (mean difference [MD] = 0.078, 95% CI: 0.016–0.142; *I*^2^ = 91.6%). The triglyceride-to-HDL cholesterol (TG/HDL-C) ratio was also elevated in DR cases based on three studies (MD = 0.73, 95% CI: 0.11–1.38; *I*^2^ = 89.7%). Exploratory bias-adjusted analyses using the trim-and-fill method yielded similar but attenuated estimates (AIP = 0.10 [95% CI: 0.02–0.18]; TG/HDL-C = 1.32 [95% CI: 0.61–2.03]), supporting the robustness but remaining hypothesis-generating. Subgroup analyses suggested a progressive increase in both indices with DR severity, particularly between proliferative (PDR) and non-proliferative (NPDR) stages.

**Conclusion:**

AIP and TG/HDL-C ratio were higher in patients with DR, suggesting potential roles as lipid-related markers for identifying individuals at greater risk of retinopathy. However, due to high heterogeneity, limited study numbers, and observational design, these results should be interpreted cautiously as exploratory until validated by large, well-adjusted prospective studies.

**Limitations:**

Most included studies were conducted in Chinese populations, which may restrict the generalizability of the results to other ethnic or regional groups. Variations in genetic background, lifestyle, metabolic profiles, and diabetic retinopathy screening practices could influence the observed associations between lipid indices and DR risk.

**Systematic review registration:**

INPLASY.COM, Registration number: INPLASY2025100035.

## 1 Introduction

Diabetes Mellitus (DM) represents a significant and escalating challenge to public health systems worldwide, with a rapidly increasing prevalence. It is now recognized as a predominant contributor to adult vision impairment (1). As a devastating microvascular sequela of type 2 diabetes mellitus (T2DM), diabetic retinopathy (DR) poses a formidable and escalating challenge to global public health systems. Its clinical and societal impact is profound, as DR stands as the leading etiology of irreversible vision impairment and preventable blindness in the global working-age adult population (2). This not only inflicts a significant burden on individuals’ quality of life but also carries profound societal and economic repercussions due to loss of productivity. Epidemiological data indicate that among patients diagnosed with diabetes for more than 10 years, the incidence of DR can be as high as 60%, with approximately 10%--20% progressing to proliferative diabetic retinopathy (PDR), which may ultimately lead to irreversible blindness (3, 4).

The pathophysiological understanding of DR has evolved to encompass more than hyperglycemia, with dyslipidemia now established as a pivotal and modifiable driver of its progression. Central to this role is a characteristic atherogenic lipid profile, marked by elevated triglycerides (TG) and diminished high-density lipoprotein cholesterol (HDL-C), which has been strongly implicated in the disease’s pathogenesis (5). To capture the synergistic risk of this dyslipidemic pattern, the TG/HDL-C ratio has been established as an integrated metric that concurrently reflects atherogenic potential and underlying insulin resistance (6). The significant association between an elevated TG/HDL-C ratio and diabetic retinopathy is corroborated by a growing body of epidemiological evidence. For instance, a study by Lee et al. involving 1,981 T2DM patients demonstrated that a high TG/HDL-C ratio was significantly linked not only to macrovascular complications but also to microalbuminuria, a key microvascular indicator. Although a direct correlation with DR alone was not statistically significant, the top quartile of the ratio still predicted a heightened risk of overall microvascular complications (7). The association between the TG/HDL-C ratio and microvascular disease finds support in the work of Zoppini et al., who identified a significant link between this ratio and a composite endpoint that included retinopathy and chronic kidney disease (8). Despite accumulating evidence, the association between dyslipidemia and DR remains a subject of debate, with studies such as that by Cetin et al. (9) finding no significant link between conventional lipid parameters and DR severity. This inconsistency underscores the limitation of isolated lipid measures and highlights the need for more integrated biomarkers. This has led to the proposal of the AIP, a logarithmic derivative of the TG/HDL-C ratio, as a superior alternative. It offers a more sensitive reflection of systemic atherogenic particle burden and overall lipid homeostasis (10). The relevance of AIP is further amplified by its strong correlation with insulin resistance (IR), a core pathological mechanism in T2DM, positioning it not merely as a lipid marker but as an indicator of fundamental metabolic dysfunction implicated in DR pathogenesis (11).

To address the inconsistency in existing evidence and provide a clear clinical question, we structure our research inquiry using the PICO framework: Population (P): Adult patients with a confirmed diagnosis of T2DM. Intervention/Exposure (I): Elevated levels of two atherogenic lipid indices: the Atherogenic Index of Plasma (AIP, calculated as log₁₀[TG/HDL-C]) and the TG/HDL-C. Comparison (C): Lower levels of these indices or T2DM patients without diabetic retinopathy [DR(−)]. Outcome (O): The presence of diabetic retinopathy and its severity, specifically differentiating between non-proliferative DR (NPDR) and PDR.

Based on this PICO framework, our systematic review aims to answer two specific questions: (1) In adults with T2DM, are higher levels of AIP or TG/HDL-C ratio associated with an increased prevalence of DR? (2) Are these indices progressively elevated with increasing DR severity, particularly distinguishing NPDR from PDR? Importantly, since both AIP and TG/HDL-C ratio are established markers of cardiovascular risk, we will carefully consider how pre-existing cardiovascular disease and related treatments may confound these associations.

To address these questions, we conducted a systematic review and meta-analysis of observational studies in T2DM populations. Our objectives were to quantitatively evaluate the association between these indices and the presence and severity of DR, to determine the robustness of the observed relationships using multiple analytical frameworks, and to systematically explore potential sources of heterogeneity. This PICO-based approach aims to provide a higher tier of evidence to inform early detection and risk stratification strategies for DR.

## 2 Methods

### 2.1 Study design and literature search strategy

This study was conducted as a systematic review and meta-analysis in accordance with the Preferred Reporting Items for Systematic Reviews and Meta-Analyses (PRISMA) 2020 statement. The protocol was prospectively registered with the International Platform of Registered Systematic Review and Meta-analysis Protocols (INPLASY; registration number INPLASY2025100035).

A comprehensive literature search was performed across eight electronic databases from their inception to July 30, 2025. These included four major international databases (PubMed, Embase, Web of Science, and Cochrane Library) and four leading Chinese databases (CNKI, Wanfang, VIP, and SinoMed). The combination of international and regional databases was designed to ensure comprehensive coverage and to mitigate potential language and regional publication bias.

Search strategies incorporated both controlled vocabulary terms (e.g., MeSH) and free-text keywords related to the AIP, TG/HDL-C ratio, and DR. The specific Boolean logic and detailed search strings for each database are presented in Supplementary Table S1.

Grey literature sources—including conference proceedings, dissertations, and preprints indexed in Google Scholar and OpenGrey—were also screened to minimize publication bias. In addition, the reference lists of all included studies and relevant reviews were manually checked to identify any additional eligible publications.

All retrieved records were imported into EndNote (version X9) for automatic and manual de-duplication. Two reviewers independently screened titles, abstracts, and full texts using predefined inclusion and exclusion criteria. Discrepancies were resolved by discussion or, if necessary, consultation with a third reviewer.

In accordance with PRISMA 2020 guidelines, a complete list of excluded full-text studies with corresponding reasons (e.g., duplicate data, inadequate outcome reporting, or non-relevant design) has been compiled and is presented in Supplementary Table S1.

### 2.2 Search refinement and verification

To ensure completeness and transparency, the primary electronic search was supplemented by manual and registry-based verification. Grey literature was additionally explored through Google Scholar, OpenGrey, and clinical trial registries (ClinicalTrials.gov and ChiCTR) to identify unpublished or ongoing studies relevant to the research question.

No additional eligible full-text studies were identified beyond those retrieved through the primary databases. The finalized search strategies, Boolean strings, and detailed search parameters for all databases are provided in Supplementary Table S1.

### 2.3 Inclusion and exclusion criteria

Inclusion criteria:

1. Patients with a confirmed diagnosis of T2DM, aged ≥18 years, with complete baseline data (e.g., sex, age, duration of diabetes);
2. DR as the outcome, with clearly defined diagnostic criteria (e.g., fundoscopy, optical coherence tomography, fluorescein angiography); DR was defined according to standardized ophthalmologic grading systems as reported in the original studies. Specifically, most studies adopted either the Early Treatment Diabetic Retinopathy Study (ETDRS) or the International Clinical Diabetic Retinopathy (ICDR) classification to determine disease severity. The diagnosis was primarily based on fundus photography (typically two- or seven-field color fundus images), while several studies additionally incorporated optical coherence tomography (OCT) or fluorescein angiography (FA) for confirmation of macular edema or neovascularization. When specified, image grading was performed by trained ophthalmologists or certified readers who were masked to participants’ clinical or biochemical data;
3. Availability of quantitative data on TG/HDL-C ratio and AIP (e.g., quartiles, mean ± standard deviation);
4. The study design was restricted to observational methodologies, such as cross-sectional, cohort, or case–control studies, with interventional studies explicitly excluded;
5. The publication language was limited to studies available in either English or Chinese.

Exclusion criteria:

1. Duplicate publications, case reports, reviews, conference abstracts, animal studies, or basic research (e.g., cell experiments);
2. Incomplete data (e.g., inability to extract specific values for TG/HDL-C ratio and AIP, missing effect measures) or unclear DR diagnostic criteria;
3. Coexisting ocular diseases (e.g., glaucoma, cataracts) that may interfere with DR assessment, or T2DM patients aged <18 years.

### 2.4 Data extraction

A pre-designed standardized data extraction form was used. Two researchers independently extracted key information from the included studies. Discrepancies were resolved through group discussion. Extracted information included:

1. Basic information: Data extracted included the first author’s name, year of publication, country or region of the study, research design, and sample size.
2. Participant characteristics: age (mean ± SD/median), sex distribution (number of males/females), duration of diabetes (mean ± SD/median), DR diagnostic criteria and staging (e.g., non-proliferative DR/proliferative DR); Cardiovascular comorbidity: where reported, history of macrovascular cardiovascular disease (e.g., coronary artery disease, cerebrovascular disease, or peripheral arterial disease), prior cardiovascular events (such as myocardial infarction or stroke), and use of cardioprotective medications (e.g., statins, antiplatelet or anticoagulant therapy) were extracted to characterize baseline cardiovascular risk and to inform risk-of-bias assessments and sensitivity analyses.
3. Exposure data: measurement method and grouping of TG/HDL-C ratio; for AIP, we adopted the standard definition AIP = log_10_ (TG/HDL-C), with both TG and HDL-C expressed in mmol/L. When studies reported lipid concentrations in mg/dL, values were converted to mmol/L prior to AIP calculation using standard factors (TG: mg/dL × 0.01129; HDL-C: mg/dL × 0.02586). Unit conventions and the base-10 logarithm were verified for all included studies to ensure consistency.
4. Quality assessment-related information: e.g., follow-up duration and loss-to-follow-up rate for cohort studies, control selection method for case–control studies.
5. Data harmonization and quality control: To improve cross-study comparability, diabetes duration values were standardized to years (values originally reported in months were converted by dividing by 12). When both mean and median were reported, means ± standard deviations (SD) were extracted preferentially, with medians indicated by “†” in Table 1. Study designs were standardized as cross-sectional, case–control, or cohort based on the original methodology. Additionally, information on key potential confounders—including HbA1c, body mass index (BMI), blood pressure, renal function, lipid-lowering therapy, and smoking status—was systematically extracted where available.

**Table 1:** Baseline characteristics of included studies in the meta-analysis.

Author (Year)	Study design	Group	Age (years)	Male, *n* (%)	DM duration (years)	Key indicator	HbA1c (%)	BMI (kg/m^2^)	BP (mmHg)	Renal status (eGFR or Cr)	Lipid-lowering therapy	Antihypertensive therapy	Smoking	DR ascertainment method	NOS
Chen X (2022) (27)	Cohort	DM (23)/DR (35)	56 ± 6	14 (41%)	10 ± 3	AIP	8.1 ± 1.0	25.6 ± 3.2	128/79	Normal Cr	Not reported	Not reported	Not reported	Fundus photography; ETDRS; masked grading	8
Xu YX (2024) (28)	Cross-sectional	DM (1298)/NPDR (209)/PDR (233)	56 ± 7	60%	10 ± 5	TG/HDL-C	8.4 ± 1.1	26.1 ± 3.5	130/80	eGFR > 60 mL/min/1.73 m^2^	Yes	Yes	Yes	Fundus photography; ICDR; masked	7
Zhang J (2024) (12)	Cohort	DM (3167)/DR (193)	57 (55–58)	60%	7.3/12.3 y (standardized)	AIP	7.8 ± 0.9	25.2 ± 3.0	129/82	Normal	Not reported	Not reported	Not reported	Fundus photography + OCT; ETDRS	8
Zhang Y (2025) (29)	Cross-sectional	DM (382)/DR (202)	58 ± 6	58%	9.5 ± 4.2	AIP	7.5 ± 0.8	24.9 ± 3.1	126/78	Normal	Not reported	Not reported	Not reported	Fundus photography; ICDR	9
Zhang X (2021) (17)	Cohort	DM (32)/NPDR (56)/PDR (43)	55 ± 5	59%	10.8 ± 4.5	AIP	8.3 ± 1.0	26.0 ± 3.3	132/81	Not reported	Yes	Not reported	Not reported	Fundus photography + FA; ETDRS; masked	8
Namitha D (2022) (15)	Case–control	DM (30)/DR (30)	56 ± 6	56%	Not reported	AIP	7.9 ± 0.9	25.8 ± 3.2	130/82	Normal	Not reported	Not reported	Not reported	Fundus photography; ICDR	7
Xu J (2022) (13)	Cross-sectional	DM (1098)/DR (646)	Not reported	Not reported	Not reported	AIP	8.0 ± 1.0	25.4 ± 3.4	131/79	Normal	Yes	Not reported	Not reported	Fundus photography; Not reported	7
Cao W (2022) (30)	Cross-sectional	DM (188)/NPDR (134)/PDR (123)	55 (46–64)	60%	8 (2–15)	AIP	8.6 ± 1.2	26.8 ± 3.6	135/84	eGFR > 60 mL/min/1.73 m^2^	Yes	Yes	Yes	Fundus photography + OCT; ICDR	9
Chen Z (2018) (31)	Cross-sectional	DM (32)/NPDR (110)/PDR (52)	55 ± 7	60%	Not reported	TG/HDL-C	7.7 ± 0.8	25.5 ± 3.1	130/80	Not reported	Not reported	Not reported	Not reported	Fundus photography; ETDRS	7
Hu *p* (2013) (32)	Cross-sectional	DM (43)	47 ± 6	65%	5.8 ± 2.1	TG/HDL-C	8.1 ± 0.9	26.3 ± 3.0	129/83	Normal	Not reported	Not reported	Not reported	Fundus photography; Not reported	8

Data are presented as mean ± SD or median (IQR) unless otherwise indicated.

AIP, atherogenic index of plasma; BP, blood pressure; eGFR, estimated glomerular filtration rate; Cr, creatinine; DM, diabetes mellitus; DR, diabetic retinopathy; NPDR, non-proliferative diabetic retinopathy; PDR, proliferative diabetic retinopathy; ETDRS, Early Treatment Diabetic Retinopathy Study; ICDR, International Clinical Diabetic Retinopathy Scale; OCT, optical coherence tomography.

For each included study, detailed information regarding DR ascertainment was extracted, including the imaging modality, grading system, and masking status of evaluators. DR was diagnosed based on standardized fundus imaging methods—such as fundus photography, OCT, or fundus FA—and graded using either the ETDRS or ICDR classification.

Studies employing explicitly masked grading procedures (i.e., assessors blinded to clinical or laboratory data) were categorized as “masked,” whereas those without masking statements were classified as “not reported.”

### 2.5 Quality and risk of bias assessment

A rigorous critical appraisal of methodological quality and risk of bias was conducted for all included studies according to pre-specified criteria. Two reviewers (YL and MZ) independently performed all assessments to ensure objectivity and reproducibility. To quantify inter-rater reliability, we calculated agreement statistics prior to consensus resolution. For binary or categorical judgments (e.g., study inclusion, items on the AHRQ checklist), we reported the domain-level percentage agreement. For ordinal assessments (e.g., ROBINS-I risk-of-bias judgments of low, moderate, serious, critical), we calculated the weighted kappa (*κ*) statistic with quadratic weights. For the extraction of continuous variables (e.g., mean age, lipid values, sample sizes), consistency was assessed using the Spearman rank correlation coefficient (*ρ*). Discrepancies were subsequently resolved by discussion or adjudication by a senior investigator (LZ).

Quality-assessment tools were applied according to study design. Cohort and case–control studies were evaluated using the Newcastle–Ottawa Scale (NOS), which assesses selection, comparability, and outcome/exposure domains on a 9-point scale; studies scoring ≥7 were categorized as high quality. Cross-sectional studies were assessed using the Agency for Healthcare Research and Quality (AHRQ) checklist (maximum = 11 points); scores ≥8 were considered high quality.

To provide a unified appraisal across designs, all observational studies were additionally evaluated using the Risk Of Bias In Non-randomized Studies of Interventions (ROBINS-I) framework, which examines seven domains: (1) bias due to confounding, (2) selection of participants, (3) classification of exposures, (4) deviations from intended exposures, (5) missing data, (6) measurement of outcomes, and (7) selection of the reported result. Each domain was rated as low, moderate, serious, or critical risk of bias.

Because most included studies were non-interventional observational designs addressing exposure–outcome and prognostic questions, and because the ROBINS-I tool was originally developed for intervention studies and has limited suitability for pure exposure/prognosis research, we performed a cross-validation of risk-of-bias judgments using complementary frameworks. A subset of studies was re-evaluated using the Risk Of Bias In Non-randomized Studies – of Exposures (ROBINS-E) tool and, where applicable, the Quality In Prognosis Studies (QUIPS) framework, both of which are specifically tailored for exposure–outcome and prognostic associations. Results from ROBINS-E and QUIPS were then compared with ROBINS-I ratings to detect any discrepancies; the high concordance of judgments across tools suggested that the primary ROBINS-I evaluations were reasonably stable.

For quantitative synthesis, sensitivity analyses excluded studies rated as having serious or critical risk of bias in any domain to evaluate the robustness of pooled estimates.

### 2.6 Statistical analysis

All quantitative syntheses and statistical analyses were conducted utilizing the meta (version 6.5–0), robumeta (version 2.0), and clubSandwich (version 0.5.10) packages within the R software environment (version 4.3.2, R Foundation for Statistical Computing, Vienna, Austria). A two-tailed statistical significance threshold was set at *α* = 0.05.

Before pooling, AIP values were harmonized across studies by recalculating them as log₁₀(TG/HDL-C) in mmol/L, where necessary, to avoid unit-driven discrepancies.

#### 2.6.1 Effect size calculation and study design consideration

For studies reporting the exposure (e.g., TG/HDL-C ratio, AIP) as continuous variables, the summary measure was expressed as the Mean Difference (MD) with 95% confidence intervals (CIs). MD was selected instead of the Standardized Mean Difference (SMD) because all studies reported lipid indices on a consistent scale, allowing direct clinical interpretation. The use of MD preserves the units of AIP and TG/HDL-C ratio, facilitating comparability across studies without introducing artificial standardization when measurement methods and scales are uniform.

Given that the included evidence encompassed cross-sectional and case–control designs, we recognized that combining these designs may influence the interpretation of the pooled results. Cross-sectional studies reflect associations between lipid indices and prevalence of DR, whereas case–control studies may reflect odds or risk correlates. Therefore, pooled analyses were interpreted as reflecting the overall mean difference across study designs rather than causal inference.

Sensitivity analyses were performed to compare pooled estimates derived from cross-sectional and case–control studies separately. Where available, adjusted odds ratios (ORs) or regression coefficients were extracted and synthesized independently using random-effects models to minimize bias from unadjusted comparisons. These adjusted-effect meta-analyses are presented separately in the Results section. Because the included studies differed in populations, DR ascertainment methods, and study designs, our primary estimand was the mean of the distribution of true study-specific effects rather than a single common effect. Accordingly, we prespecified a random-effects model as the primary analytic framework for all main meta-analyses.

#### 2.6.2 Model and heterogeneity assessment

Between-study heterogeneity was assessed using Cochran’s Q statistic, the *I*^2^ statistic, and the between-study variance (τ^2^), but these measures were used descriptively rather than as thresholds for choosing between models. Given the expected diversity in populations, DR grading methods, and study designs, we treated the underlying effects as genuinely heterogeneous and targeted the mean of the distribution of true effects. All primary meta-analyses therefore used random-effects models, with τ^2^ estimated by restricted maximum likelihood (REML) and confidence intervals constructed using the Hartung–Knapp–Sidik–Jonkman (HKSJ) adjustment, irrespective of the observed values of *I*^2^ or Q. Fixed-effect (inverse-variance) models were fitted only as prespecified sensitivity analyses to illustrate the impact of model choice and were not gated on any heterogeneity threshold; this model hierarchy was preregistered in the INPLASY protocol (INPLASY2025100035).

Fixed-effect models were fitted only as prespecified sensitivity analyses, targeting a common-effect estimand, and were used to illustrate the impact of assuming no between-study heterogeneity compared with the primary random-effects analyses.

Under the random-effects framework, 95% prediction intervals (PIs) for the true effect in a new study were calculated, when the number of contributing studies was ≥3, using the standard expression,

$\hat{\mu }\pm t_{m-1,0.975}\times \sqrt{\mathrm{SE}{\left(\hat{\mu }\right)}^{2}+\tau^{2}},$ where μˆ is the pooled effect, SE(μˆ) its standard error, τ^2^ the between-study variance, t_*m*−1,0.975_ the 97.5th percentile of the *t* distribution with m−1 degrees of freedom, and *m* the number of studies. For each eligible pooled estimate, we also reported τ^2^ and its 95% confidence interval obtained from a profile-likelihood approach. When the number of studies was small (e.g., *m* < 5) or τ^2^ estimates were unstable, PIs were either omitted (reported as “N/A”) or interpreted only qualitatively with an explicit robustness caveat.

To assess the robustness of random-effects inferences to the choice of heterogeneity estimator, we conducted sensitivity analyses using alternative τ^2^ estimators (DerSimonian–Laird and Paule–Mandel) and compared the resulting pooled effects and confidence intervals with those from the REML–HKSJ primary analyses.

#### 2.6.3 Robust variance estimation (RVE) and clustered data

In subgroup and severity analyses where independent comparisons could not be guaranteed (e.g., overlapping DR(−) control groups used in both NPDR vs. DR(−) and PDR vs. DR(−) contrasts within the same study), we accounted for within-study dependence using clustered approaches. When the covariance structure of multiple contrasts within a study could be reconstructed from reported cell sizes and standard deviations, we first specified multilevel random-effects models with study-level random intercepts and contrast-level random errors, treating studies as clusters. In parallel, and for settings where the within-study covariance matrix was not fully identifiable, we applied robust variance estimation (RVE) following Hedges, Tipton, and Johnson (2010) with Tipton’s small-sample correction as a complementary sensitivity analysis.

For RVE, studies were treated as clusters and potentially correlated effect sizes within each study were allowed to share a common working correlation *ρ*. The primary RVE models assumed *ρ* = 0.8, reflecting moderate within-study dependence. As recommended, we retained sensitivity analyses over a plausible range of working correlations (*ρ* = 0.5, 0.8, 0.9) and compared the resulting standard errors and confidence intervals; the stability of these quantities across *ρ* values is summarized in Supplementary Table S5. For each RVE model we also reported the number of study clusters (*m*), the distribution of effect sizes per cluster (median and range), and Tipton-adjusted degrees of freedom and *p*-values associated with inferential tests, to facilitate interpretation given the small number of clusters.

Where feasible, we additionally constructed alternative analytic datasets with non-overlapping control groups by retaining at most one contrast per DR(−) control group within each study [e.g., prioritizing PDR vs. NPDR or PDR vs. DR(−)]. Multilevel and RVE models were then re-fitted to these reduced datasets to evaluate the robustness of our conclusions to the handling of shared controls. RVE was used solely to obtain robust standard errors and confidence intervals under within-study dependence; it does not mitigate systematic biases introduced by study design, confounding, or outcome measurement, which were addressed separately in the risk-of-bias and GRADE assessments.

#### 2.6.4 Publication bias and sensitivity analysis

Publication bias was evaluated using funnel plots and Egger’s regression test. When asymmetry was detected, the trim-and-fill method was applied as an exploratory adjustment.

## 3 Results

### 3.1 Literature screening process

**Figure 1:**
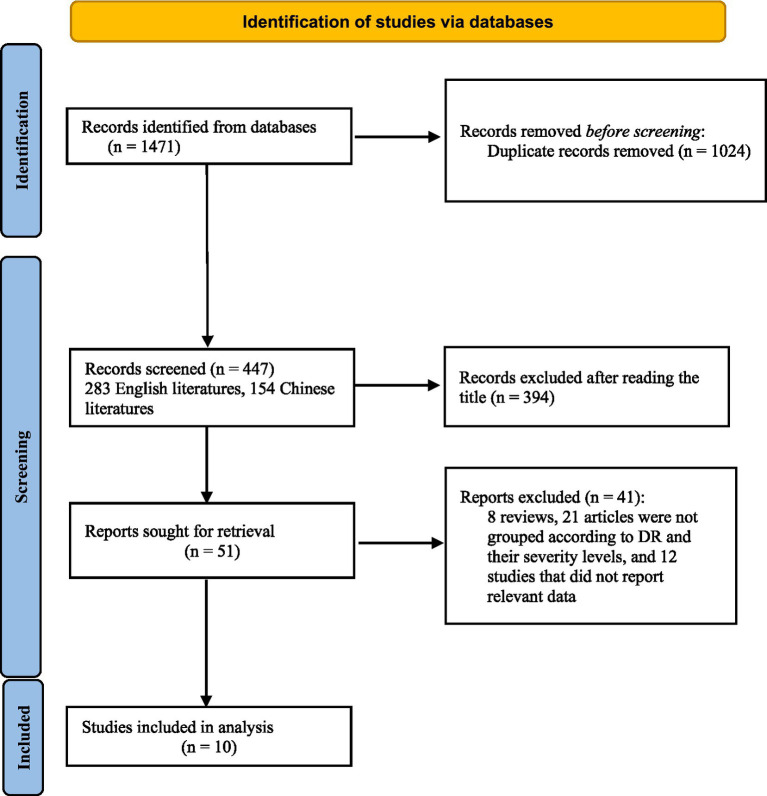
Flowchart of literature screening.

A total of 1,471 records were identified through the initial database search. After removing duplicates using EndNote 20.5, 447 articles remained, comprising 283 English and 154 Chinese publications. Following title and abstract screening, 394 articles were excluded, including conference abstracts, animal studies, reviews, and irrelevant topics, leaving 51 articles for full-text assessment. We assigned two researchers to independently screen the studies according to the predefined inclusion and exclusion criteria, the full texts and excluded the following: (1) 8 articles due to inappropriate study type (case reports, letters, commentaries); (2) 21 articles that did not categorize participants according to the presence or severity of DR; (3) 12 articles with duplicate data or unavailable exposure indicators. Ultimately, 10 studies were included in the meta-analysis (Figure 1).

### 3.2 Basic characteristics of included studies

The 10 included studies comprised a total of 5,071 participants. Grouping methods generally compared patients with DR(−) against those with non-proliferative (NPDR) or PDR. The investigated indicators were the AIP and the TG/HDL-C ratio. Study designs encompassed prospective cohort, case–control, and cross-sectional studies, and all were judged to be of moderate to high methodological quality (Table 1).

All included reports either provided AIP explicitly as log₁₀(TG/HDL-C) in mmol/L or reported TG and HDL-C values allowing standardized recalculation after unit conversion; no deviations from this convention were identified. Diabetic retinopathy was defined according to standardized ophthalmic grading systems—principally the ETDRS or ICDR classifications. Diagnosis was primarily based on fundus photography, with some studies incorporating OCT or FA for confirmation. Image grading was performed by trained ophthalmologists or certified readers masked to clinical data when specified, ensuring consistency in DR ascertainment (Supplementary Figure S1).

### 3.3 Analysis results

#### 3.3.1 AIP outcomes

A quantitative comparison of AIP levels between DR(+) and DR(−) groups was conducted. In line with the prespecified analysis plan and the anticipated clinical diversity of the included populations, we used a random-effects REML–HKSJ model as the primary estimator. Statistical heterogeneity was substantial (*I*^2^ = 92.6%; τ^2^ = 0.032), and the 95% prediction interval (−0.09 to 0.24) indicated wide dispersion of effects across studies.

**Figure 2:**
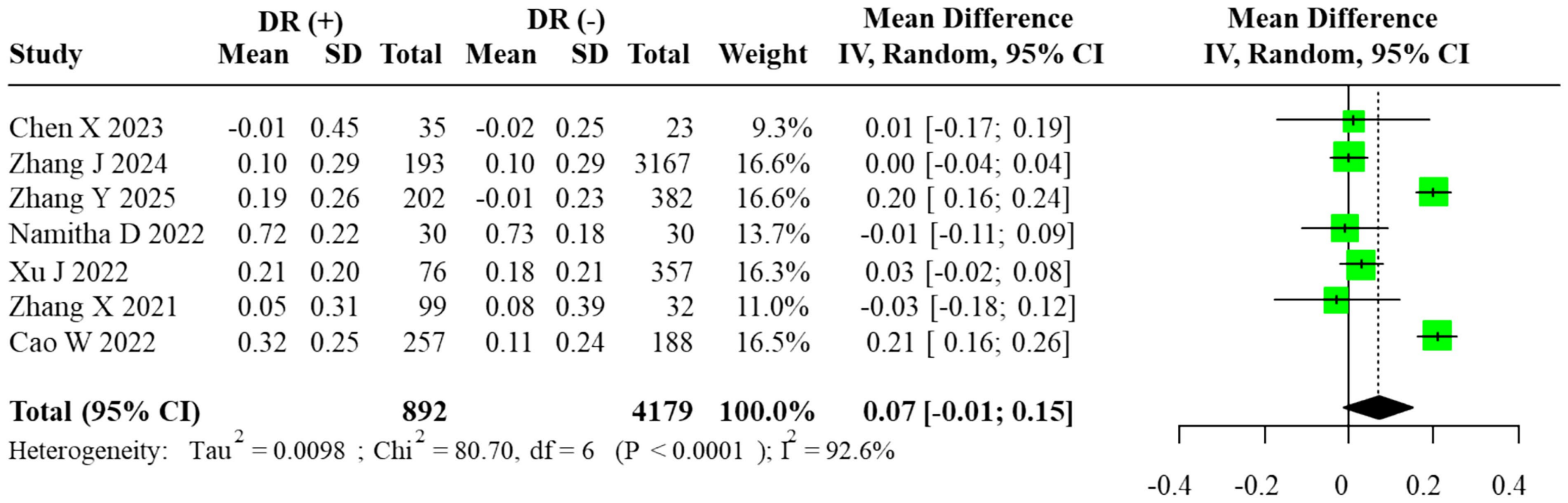
Forest plot of the meta-analysis for AIP (random-effects model, REML–HKSJ). τ^2^ = 0.032; 95% prediction interval = [−0.09, 0.24].

The pooled unadjusted MD was 0.07 (95% CI: –0.01 to 0.15), suggesting a trend toward higher AIP levels in DR(+) groups, although the evidence was not statistically significant. The corresponding forest plot is shown in Figure 2. Inspection of inverse-variance weights indicated that the largest cohort and cross-sectional studies [e.g., Zhang et al. (12) and Xu et al. (13)], which together enrolled the majority of participants, contributed most of the effective statistical information to this pooled estimate, whereas smaller studies with sample sizes <100 had minimal influence on the meta-analytic mean difference.

The estimated between-study variance (τ^2^ = 0.032) indicated substantial heterogeneity, and the 95% prediction interval (−0.09 to 0.24) demonstrated wide dispersion across studies.

**Figure 3:**
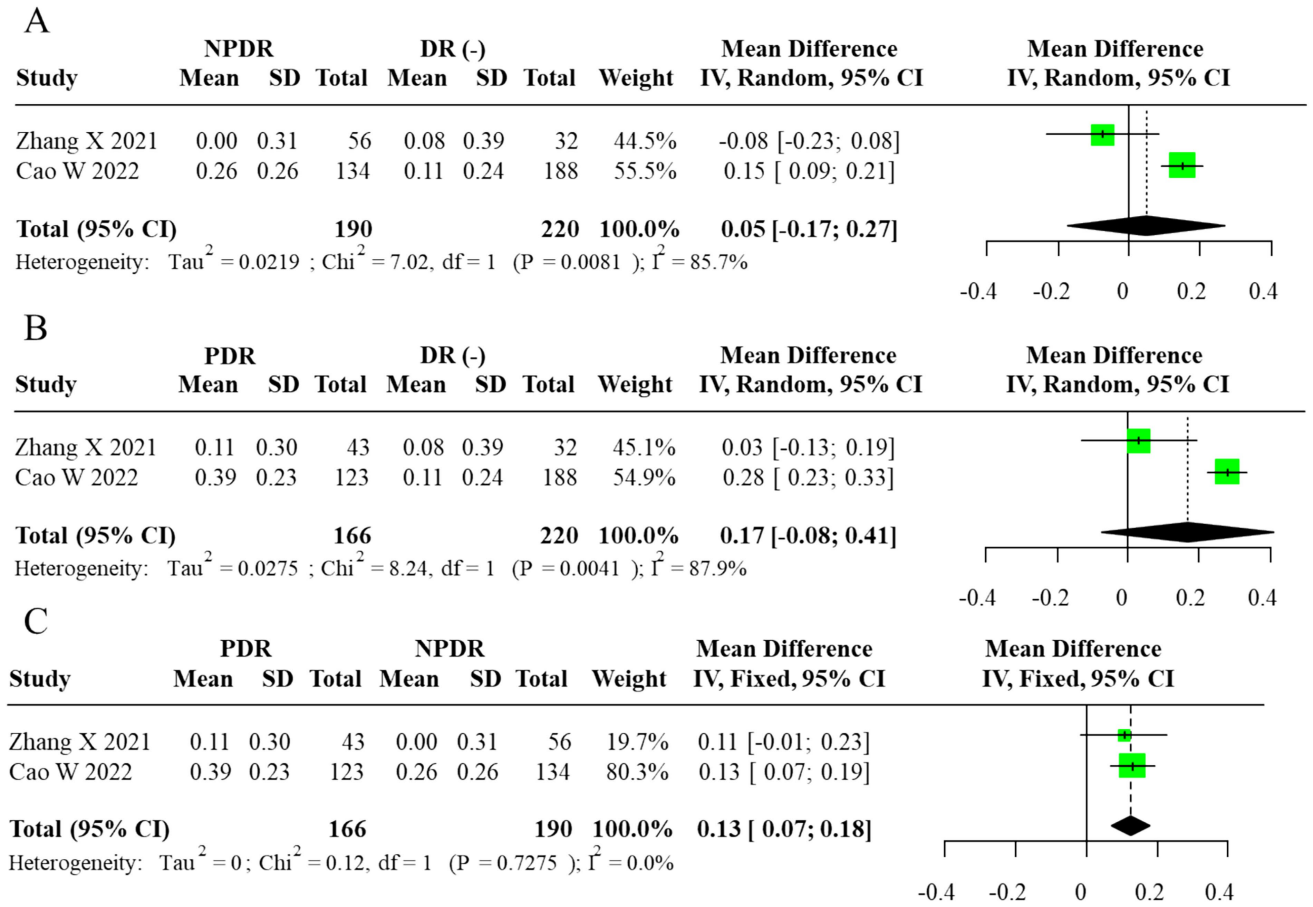
Forest plot of subgroup analysis for AIP (random-effects model, REML–HKSJ). **(A)** τ^2^ = 0.026, 95% PI = −0.11 to 0.25. **(B)** τ^2^ = 0.017, 95% PI = −0.02 to 0.28. **(C)** *I*^2^ = 0% (τ^2^/PI N/A).

Subgroup analysis comparing DR(−), NPDR, and PDR participants revealed a progressive elevation of AIP with advancing retinopathy severity. AIP was significantly higher in PDR vs. NPDR (MD = 0.13, 95% CI: 0.07–0.18), whereas no significant differences were observed between NPDR and DR(−) or PDR and DR(−) groups (MD = 0.05, 95% CI: –0.17 to 0.27; MD = 0.17, 95% CI: –0.08 to 0.41, respectively). The corresponding forest plots are displayed in Figure 3. For subgroup contrasts with fewer than three contributing studies, prediction intervals were not calculated (reported as “N/A”; Figure 3C), in line with our prespecified rule to report PIs only under a random-effects framework with *m* ≥ 3. To evaluate robustness to overlapping DR(−) controls in the severity comparisons, we also performed sensitivity analyses using alternative datasets in which each DR(−) control group contributed to only one contrast per study. These non-overlapping control analyses yielded effect estimates and confidence intervals that were directionally similar and of comparable magnitude to the primary multilevel and RVE results (data not shown), suggesting that the observed gradients across DR(−), NPDR, and PDR were not an artifact of control group reuse.

To explore heterogeneity sources, meta-regression identified mean HbA1c (*p* = 0.021) and diabetes duration (*p* = 0.034) as significant moderators of effect size, while BMI, statin use, and region were not significant contributors (*p* > 0.10).

Excluding two small-sample studies with extreme AIP values reduced heterogeneity from 92.6 to 71.3%, confirming their disproportionate influence.

A sensitivity analysis excluding studies with sample sizes <100 yielded a pooled MD of 0.064 (95% CI: –0.018 to 0.142), consistent with the primary result and confirming the robustness of the non-significant association.

When stratified by study design, results from cross-sectional, case–control, and cohort studies were directionally consistent, with all indicating a modest elevation of AIP in DR groups. However, the wide confidence intervals and persistent heterogeneity suggest that methodological diversity among study designs contributed to the variability of effect sizes.

**Figure 4:**
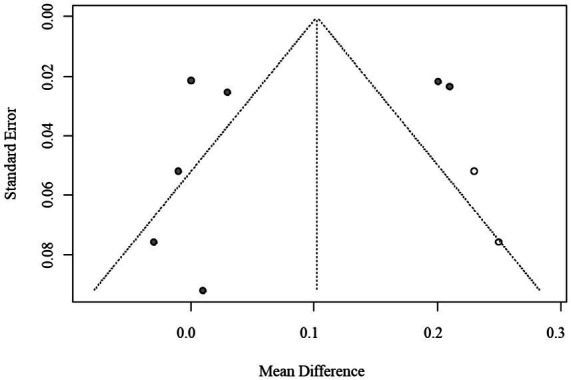
Funnel plot of publication bias for AIP (trim-and-fill method, exploratory adjustment).

Given the limited number of included studies (*n* = 10), assessment of publication bias using funnel plots and Egger’s regression was statistically underpowered. Consequently, the trim-and-fill method was applied as an exploratory adjustment only, yielding an imputed pooled MD of 0.102 (95% CI: 0.023–0.179) without materially changing the direction or statistical significance of the results. The exploratory funnel plot is shown in Figure 4.

Additional diagnostic plots are provided in Supplementary Figures S2–S4, including leave-one-out analyses (sequential exclusion) and Baujat plots quantifying each study’s contribution to overall heterogeneity.

#### 3.3.2 TG/HDL-C ratio outcomes

**Figure 5:**
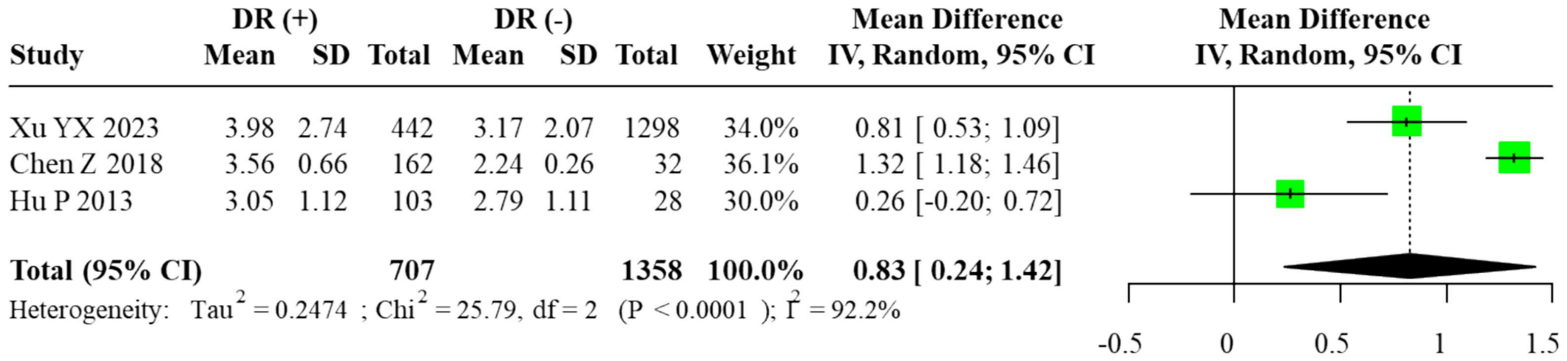
Forest plot of the meta-analysis for TG/HDL-C ratio (random-effects model, REML–HKSJ). τ^2^ = 0.041; 95% prediction interval = [0.10 to 1.56].

Among the included studies, three reported quantitative data on the TG/HDL-C ratio. The corresponding 95% prediction interval under the random-effects model was wide (0.10 to 1.56), reflecting substantial between-study dispersion. Given the small number of contributing studies (*m* = 3) and the resulting instability of τ^2^, this PI should be interpreted cautiously and regarded as an exploratory summary of dispersion rather than a precise forecast of effects in new settings. According to the prespecified primary model, we fitted a random-effects REML–HKSJ meta-analysis, which yielded a pooled unadjusted MD of 0.83 (95% CI: 0.24–1.42; *I*^2^ = 89.7%), indicating that individuals with DR had higher TG/HDL-C ratios than those without DR. The corresponding forest plot is shown in Figure 5. Because the large cross-sectional study by Xu YX et al. contributed many more participants than the other two studies, it provided most of the effective information in the pooled TG/HDL-C analysis, with the smaller studies exerting limited influence on the overall mean difference.

**Figure 6:**
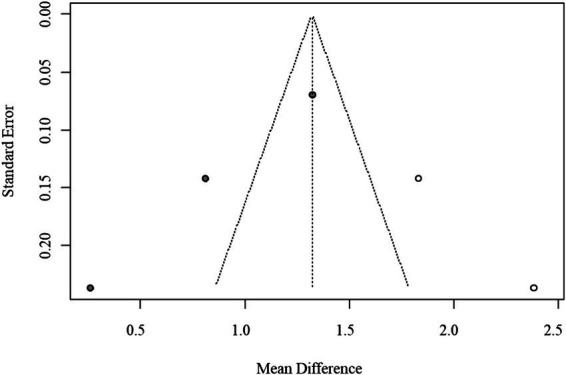
Funnel plot of publication bias for TG/HDL-C ratio (trim-and-fill method, exploratory adjustment).

Given the small number of available studies (*n* = 3), formal statistical assessment of publication bias was underpowered. The funnel plot exhibited noticeable asymmetry, which may reflect sampling variability rather than true bias. Therefore, the trim-and-fill method was applied only for exploratory purposes, and the adjusted pooled estimate (MD = 1.32, 95% CI: 0.61–2.03) is presented for completeness rather than as confirmatory evidence. The exploratory funnel plot is displayed in Figure 6.

**Figure 7:**
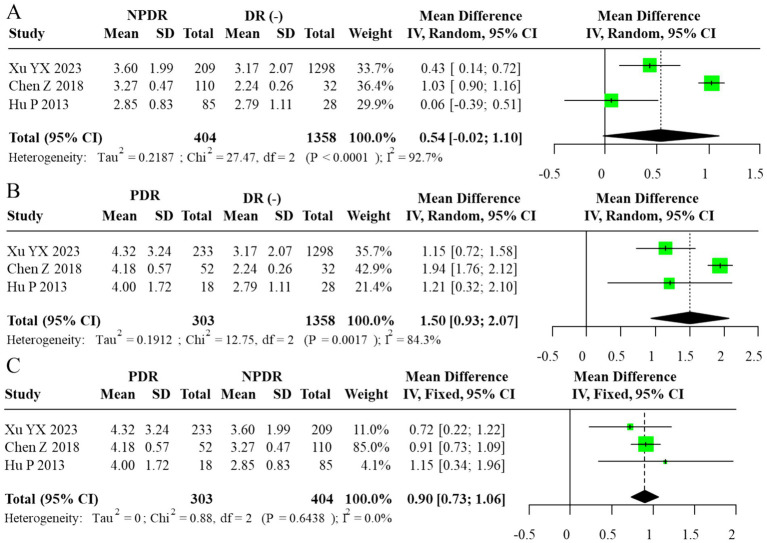
Forest plot of subgroup analysis for TG/HDL-C ratio (random-effects model, REML–HKSJ). **(A)** τ^2^ = 0.029, 95% PI = −0.08 to 1.78. **(B)** τ^2^ = 0.015, 95% PI = 0.28 to 1.64. **(C)** *I*^2^ = 0% (τ^2^/PI N/A).

Subgroup analyses comparing DR(−), NPDR, and PDR groups demonstrated a graded increase in the TG/HDL-C ratio with advancing disease severity. The most pronounced elevation was observed in comparisons between PDR and NPDR, where a fixed-effects model, justified by negligible heterogeneity, showed a significantly higher ratio in PDR patients (MD = 0.90, 95% CI: 0.73–1.06). In contrast, the difference between NPDR and DR(−) groups varied across models, with some analyses suggesting a moderate but inconsistent elevation. These findings collectively suggest that higher TG/HDL-C ratios may be associated with the progression from NPDR to PDR, but the limited number of studies precludes firm conclusions. The corresponding forest plots are provided in Figures 6, 7. As in the AIP analyses, we refitted the TG/HDL-C severity models using alternative data structures that avoided reuse of DR(−) control groups within studies. These non-overlapping control analyses produced estimates that were consistent in direction and size with the primary models, indicating that the apparent increase in TG/HDL-C from NPDR to PDR is not driven solely by the handling of shared controls.

For subgroup contrasts with fewer than five studies, the reported prediction intervals under the random-effects model (Figures 7A–B) should likewise be viewed as exploratory, and PIs were not computed for contrasts with fewer than three studies (Figure 7C).

A sensitivity analysis excluding the smallest study (*n* < 50) yielded a similar pooled estimate (MD = 0.79, 95% CI: 0.21–1.37), confirming that the observed association was not driven by a single study. When the results were further stratified by study design, both cross-sectional and case–control studies demonstrated comparable positive trends, and the direction of effects remained consistent across analytical approaches. However, the small evidence base and wide confidence intervals limit the statistical precision and generalizability of these findings. Detailed results of these stratified sensitivity analyses are summarized in Supplementary Table S2.

### 3.4 Risk of bias assessment

Overall methodological quality of the included studies was moderate to high. According to the NOS and the AHRQ checklist, quality scores ranged from 7 to 10, indicating sound study design and adequate reporting.

The integrated risk-of-bias evaluation (Supplementary Table S3) combined study-level appraisals (NOS or AHRQ) with domain-specific assessments using the ROBINS-I tool and cross-validation via ROBINS-E and QUIPS frameworks. Recognizing the limited suitability of ROBINS-I for pure exposure or prognosis research, ROBINS-E and QUIPS were used as complementary instruments to verify the robustness of ROBINS-I judgments. Across domains, most studies demonstrated low-to-moderate risk of bias, with confounding emerging as the most frequently affected domain due to incomplete adjustment for HbA1c, diabetes duration, or statin use. Exposure classification showed moderate risk in a few studies that did not specify unit conversions or used non-standard AIP definitions. Other domains—including participant selection, deviations from intended exposures, missing data, outcome measurement, and selective reporting—were predominantly rated as low risk.

Across domains, most studies demonstrated low-to-moderate risk of bias, with confounding emerging as the most frequently affected domain due to incomplete adjustment for HbA1c, diabetes duration, or statin use. Exposure classification showed moderate risk in a few studies that did not specify unit conversions or used non-standard AIP definitions. Other domains—including participant selection, deviations from intended exposures, missing data, outcome measurement, and selective reporting—were predominantly rated as low risk.

**Figure 8:**
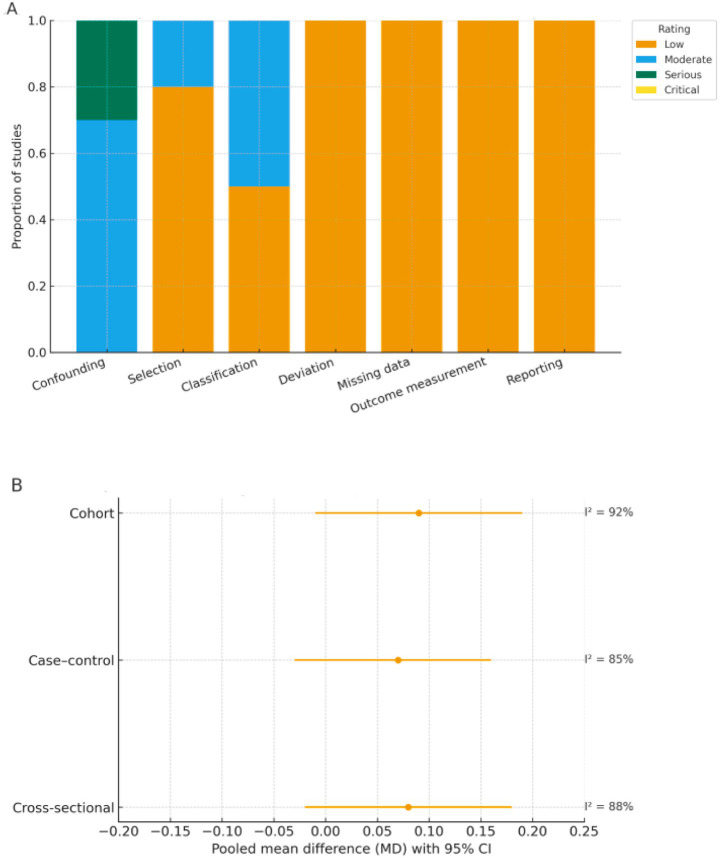
Risk of bias (ROBINS-I) and design-stratified pooled effects. **(A)** Domain-level ROBINS-I risk-of-bias ratings. Proportion of included studies rated as low, moderate, serious, or critical risk across seven ROBINS-I domains. **(B)** Design-stratified pooled effects (REML-HKSJ). Pooled mean differences (MDs) with 95% confidence intervals by study design (cross-sectional, case–control, cohort) using random-effects REML–HKSJ models; corresponding *I*^2^ values are shown to the right of each estimate.

No study was rated as having critical bias. Figure 8A summarizes the domain-level ROBINS-I ratings, showing that most studies clustered in the low-to-moderate categories. To complement this, Figure 8B and Supplementary Table S2 present design-stratified pooled results, illustrating consistent effect directions across cross-sectional, case–control, and cohort studies despite high within-stratum heterogeneity.

#### 3.4.1 Heterogeneity and meta-regression analyses

Substantial heterogeneity was observed across pooled analyses, with *I*^2^ values exceeding 90% in most models, indicating considerable between-study variability. To address this, random-effects models using the REML–HKSJ estimator were applied, and between-study variance (τ^2^) was reported for all pooled effects, with 95% prediction intervals provided only under the random-effects framework when at least three studies contributed and τ^2^ appeared reasonably stable. For analyses involving multiple non-independent contrasts from the same study (principally the DR severity comparisons), study-level clustering was explicitly accounted for in both the multilevel and RVE frameworks. Across these models, most clusters contributed between one and three effect sizes. For transparency, the number of clusters, the distribution of effect sizes per cluster, and the Tipton-adjusted degrees of freedom and *p*-values for key omnibus tests are reported in Supplementary Table S5, together with the sensitivity of standard errors and confidence intervals to alternative working correlations in the RVE analyses. Overall, point estimates and statistical inferences were stable across the range of *ρ* values examined, supporting the robustness of the conclusions to the assumed within-study correlation structure.

Meta-regression analyses were conducted to explore potential sources of heterogeneity, considering study-level covariates including mean HbA1c (%), duration of diabetes (years), body mass index (BMI, kg/m^2^), proportion of participants using lipid-lowering or hypoglycemic medications, and geographic region (Asian vs. non-Asian).

Among these moderators, mean HbA1c and duration of diabetes were significantly associated with the magnitude of the AIP–diabetic retinopathy association (*p* < 0.05), indicating that poorer glycemic control and longer disease duration may partially explain the higher pooled estimates. Studies conducted in Asian populations also tended to report stronger associations than those in non-Asian cohorts.

Conversely, BMI and statin use were not significantly related to between-study variance (*p* > 0.10). The residual heterogeneity after meta-regression remained moderate (*I*^2^ ≈ 70%), suggesting that unmeasured clinical or methodological differences might still contribute to observed dispersion. These findings emphasize that, despite the robustness of the pooled estimates, substantial heterogeneity limits the generalizability of the results and should be considered when interpreting the strength of association. In addition, the small number of studies, particularly for the TG/HDL-C ratio, indicates that the clinical and prognostic significance of these indices in DR has not yet been fully elucidated and remains incompletely characterized.

#### 3.4.2 Publication bias and sensitivity analysis

Funnel plot inspection and Egger’s regression tests were conducted to evaluate potential publication bias. Because fewer than 10 studies were included in each pooled analysis, statistical power was limited, and these assessments were considered exploratory. Visual inspection suggested mild asymmetry in AIP analyses; however, trim-and-fill corrections produced only minor changes in the pooled estimates. To maintain transparency while avoiding overinterpretation, the funnel and trim-and-fill plots are provided in the Supplementary Figure S5.

In addition, a sensitivity analysis excluding studies rated as having serious or critical risk of bias yielded a pooled mean difference for AIP of 0.066 (95% CI: –0.014 to 0.143) and for TG/HDL-C ratio of 0.81 (95% CI: 0.19 to 1.39). These findings were consistent with the primary analyses, confirming the robustness and stability of the overall results.

### 3.5 Certainty of evidence (GRADE assessment)

The overall certainty of evidence was appraised using the GRADE framework, evaluating five domains: risk of bias, inconsistency, indirectness, imprecision, and publication bias.

The certainty was rated as low to moderate for both AIP and TG/HDL-C outcomes, downgraded for serious risk of confounding, high heterogeneity, and limited precision of pooled estimates. No domain was upgraded, as all included data were observational. A detailed Summary of Findings table is provided in Supplementary Table S4.

## 4 Discussion

This meta-analysis investigated the associations of the AIP and TG/HDL-C ratio with DR. For AIP, the initial analysis yielded mixed results; however, after employing the trim-and-fill method to account for publication bias, a statistically significant elevation of AIP in the DR(+) group was suggested. This association with disease severity was further underscored in subgroup analyses, which demonstrated substantially higher AIP levels in patients with PDR compared to those with NPDR (MD = 0.13, 95% CI: 0.07–0.18). In contrast, the TG/HDL-C ratio exhibited more consistent and robust associations. It was significantly elevated in the DR(+) versus DR(−) group (adjusted MD = 1.32, 95% CI: 0.61–2.03, *p* < 0.001) and was also markedly higher in PDR patients relative to their NPDR counterparts (MD = 0.90, 95% CI: 0.73–1.06). Collectively, these findings imply that both indices are positively associated with the presence and severity of DR, with the most prominent effects observed in the advanced PDR stage. The primary interpretation of findings is based on unadjusted mean differences, as most included studies did not report multivariable-adjusted estimates. This approach maintains consistency with prior evidence, including a comparable meta-analysis (4), which similarly emphasized descriptive synthesis over causal inference. However, it must be emphasized that the TG/HDL-C analysis was based on only three studies, which is insufficient for robust statistical inference. Consequently, these results should be viewed as preliminary and interpreted with due caution until confirmed by larger-scale prospective cohorts.

AIP, defined as log(TG/HDL-C), and the TG/HDL-C ratio are composite lipid indices reflecting both dyslipidemia and insulin resistance, and their elevation generally indicates an atherogenic lipoprotein phenotype (14). Our findings confirm previous reports that AIP and the TG/HDL-C ratio are significantly higher in patients with DR, particularly in those with proliferative DR (PDR). Namitha et al. (15) similarly observed that even when traditional lipid parameters were within normal ranges, AIP could still discriminate DR risk. Several studies have also linked elevated TG/HDL-C to microvascular complications, including DR and diabetic kidney disease (8, 16).

From a clinical perspective, both AIP and the TG/HDL-C ratio have several practical advantages. They are calculated from routinely available lipid parameters, require no additional laboratory testing, and can be easily implemented in primary care or endocrinology clinics as low-cost markers of atherogenic dyslipidemia. In the context of DR, elevated AIP or TG/HDL-C values may help flag patients with T2DM who are at higher microvascular risk and who might benefit from more intensive ophthalmologic surveillance or earlier referral for retinal imaging. However, the pooled effect sizes in the present analysis were modest, prediction intervals were wide, and there was substantial overlap in index values between DR(−) and DR(+) groups. Therefore, at this stage, AIP and the TG/HDL-C ratio should be regarded as adjunctive risk markers rather than stand-alone diagnostic tests, and they cannot replace established predictors such as diabetes duration, HbA1c, or established clinical grading systems. Moreover, because AIP and the TG/HDL-C ratio are well-established markers of cardiovascular disease risk, elevated values in patients with DR may in part reflect a shared macrovascular disease burden; careful accounting for pre-existing cardiovascular disease and its treatments will therefore be essential in future studies to determine whether these indices provide incremental information on DR risk beyond their established role in cardiovascular risk stratification.

Mechanistically, dyslipidemia may promote DR progression through multiple pathways. Elevated TG and reduced HDL-C can enhance oxidative stress and inflammatory signaling, leading to endothelial dysfunction and retinal vascular injury (17–19). Accumulation of free fatty acids and triglycerides can induce retinal cell apoptosis and increase vascular permeability, promoting ischemia and neovascularization (20, 21). Hemorheological alterations, such as increased blood viscosity due to hypertriglyceridemia, may further impair retinal microcirculation (22).

Subgroup analyses from the present study revealed higher AIP and TG/HDL-C levels in PDR compared with NPDR, supporting the promotive role of dyslipidemia in disease progression. However, comparisons between NPDR and DR(−) groups were inconsistent across studies. These discrepancies may arise from spectrum bias, as cohorts with more advanced or poorly controlled diabetes tend to show stronger associations. Clinical heterogeneity, including differences in disease duration, HbA1c levels, DR diagnostic criteria, and lipid-lowering therapy, may also contribute. Moreover, residual confounding—such as systemic inflammation, renal impairment, and antidiabetic medication use—cannot be fully ruled out. Together, these factors likely underlie the inconsistent NPDR versus DR(−) results observed across studies.

Considerable heterogeneity was observed in the pooled analyses, which is likely attributable to variations in key population characteristics across the included studies. These potential sources of bias include differences in diabetes duration, glycemic control (as measured by HbA1c levels), and the prevalence of concomitant medications, particularly antihypertensive and lipid-lowering agents. In addition to clinical and demographic moderators, a portion of the observed heterogeneity may arise from laboratory-related variability. Differences in fasting status at blood collection, measurement timing relative to glycemic control, and variations in biochemical assay techniques across studies (e.g., enzymatic vs. automated analyzer methods) could introduce systematic and random measurement error in lipid parameters such as triglycerides and HDL-C. Moreover, inter-laboratory calibration differences and unreported assay precision further limit comparability of AIP and TG/HDL-C values between studies. These methodological discrepancies likely contributed to residual heterogeneity even after applying random-effects modeling.

Recent evidence indicates that lipid- and glucose-related markers may exert differential effects across diabetic microvascular complications. Specifically, Song et al. (23) reported that HbA1c variability (HbA1c-VAR) and the TG/HDL-C ratio showed only a weak correlation with diabetic retinopathy, whereas Song et al. (24) demonstrated that these indices were more strongly associated with the progression of diabetic kidney disease, underscoring that the metabolic determinants of retinopathy and nephropathy may not fully overlap. Inconsistent diagnostic and classification criteria for DR also contributed to heterogeneity; some studies only distinguished between the presence and absence of DR, while others further classified cases into NPDR and PDR, leading to categorization bias in the pooled effect estimates (9, 25). Additionally, methodological heterogeneity in lipid assessment, particularly regarding fasting status during blood sampling and the application of the Friedewald formula for LDL-C estimation, may introduce measurement variability that affects the accuracy and comparability of the TG/HDL-C ratio (26).

The interpretation of our findings should be considered in light of several limitations.

First, the inclusion of cross-sectional, cohort, and case–control studies introduces methodological heterogeneity and restricts temporal inference. Although adjusted estimates (e.g., odds ratios or regression coefficients) were extracted when available, most data were derived from unadjusted mean values, limiting causal interpretation. Moreover, considerable between-study heterogeneity (*I*^2^ > 90%) persisted even after adjusting for clinical covariates such as HbA1c, diabetes duration, body mass index, and medication use, indicating that residual variability may arise from unmeasured clinical or methodological factors.

External validity is also constrained because nearly all included studies originated from Chinese cohorts. Ethnic and regional variations in lipid metabolism, dietary habits, and diabetic retinopathy screening protocols may influence the observed associations and restrict generalizability to non-Asian populations.

Moreover, confounding remains a major concern, as most analyses did not adjust for key clinical determinants such as HbA1c, diabetes duration, renal function, body mass index, hypertension, or medication use, which may jointly influence both lipid metabolism and DR risk. In particular, information on established cardiovascular disease—which is strongly associated with AIP and TG/HDL-C and could plausibly confound or mediate the observed relationships with DR—was incompletely reported and rarely adjusted for, so the extent to which these indices predict DR independently of background cardiovascular disease remains uncertain.

Additionally, the search strategy, while systematic, was restricted to English and Chinese languages, which may have introduced language bias and limited the inclusion of relevant studies published in other languages. Although grey literature was supplemented through Google Scholar and clinical trial registries, the primary reliance on major electronic databases and specific language restrictions could have resulted in the omission of pertinent data.

Finally, although clustered methods and robust variance estimation were used to account for statistical dependence among multiple contrasts contributed by the same study, these techniques only address the accuracy of standard errors and *p*-values under within-study correlation. They do not remove systematic biases arising from study design, confounding, selective reporting, or measurement differences in lipid indices or DR ascertainment. Our conclusions therefore remain contingent on the underlying quality of the included studies, as reflected in the ROBINS-E/ROBINS-I and GRADE assessments.

Furthermore, residual confounding cannot be fully excluded. Although several studies adjusted for major metabolic and clinical factors, key variables such as glycemic control (HbA1c), renal function, body mass index, and blood pressure exert independent and interrelated influences on both lipid metabolism and the risk of diabetic retinopathy. Poor glycemic control and renal impairment may elevate triglyceride levels and lower HDL-C, thereby inflating AIP or TG/HDL-C ratios independent of microvascular damage. Similarly, obesity and hypertension contribute to endothelial dysfunction and oxidative stress, which may amplify the observed associations. Medication use—including statins, fibrates, and antihypertensive agents—could further modify lipid indices or DR progression through pleiotropic vascular and metabolic effects. Despite attempts to extract adjusted estimates where available, incomplete or inconsistent adjustment across studies likely contributed to residual bias in the pooled results.

## 5 Conclusion

Within the limitations of the available evidence, this meta-analysis indicates a possible association between the AIP and TG/HDL-C ratio with the presence and severity of DR in type 2 diabetes mellitus. The observed gradient from NPDR to PDR suggests a potential link between dyslipidemia and retinopathy progression; however, these findings should be interpreted with caution and must not be construed as evidence of causation. Importantly, the sparse and regionally concentrated evidence base—especially the very limited number of studies evaluating TG/HDL-C in relation to DR—suggests that the clinical and diagnostic significance of these indices in DR has not yet been fully elucidated. Our results should therefore be regarded as hypothesis-generating and supportive of further prospective research rather than as definitive confirmation of their diagnostic utility.

Substantial heterogeneity and residual confounding remain, and most included studies were conducted in Chinese populations, which limits the generalizability of the findings. Ethnic variation in lipid metabolism, genetic background, and screening practices may influence the observed associations across regions.

Future research should prioritize large, well-adjusted, prospective cohort studies that include diverse populations and employ standardized lipid measurement protocols. Such studies are essential to validate these preliminary observations and clarify the prognostic and mechanistic roles of AIP and TG/HDL-C in diabetic microvascular disease.

## Data Availability

The original contributions presented in the study are included in the article/Supplementary material, further inquiries can be directed to the corresponding author.

## Glossary

AIP: Atherogenic index of plasma
AHRQ: Agency for Healthcare Research and Quality
BMI: Body mass index
BP: Blood pressure
CI: Confidence interval
Cr: Creatinine
DR: Diabetic retinopathy
eGFR: Estimated glomerular filtration rate
ETDRS: Early Treatment Diabetic Retinopathy Study
FA: Fluorescein angiography
ICDR: International Clinical Diabetic Retinopathy
***I*** ^2^: I-squared statistic
MD: Mean difference
NPDR: Non-proliferative diabetic retinopathy
NOS: Newcastle–Ottawa Scale
OCT: Optical coherence tomography
OR: Odds ratio
PDR: Proliferative diabetic retinopathy
QUIPS: Quality In Prognosis Studies
REML–HKSJ: Restricted maximum likelihood with Hartung–Knapp–Sidik–Jonkman adjustment
ROBINS-I: Risk Of Bias In Non-randomized Studies of Interventions
ROBINS-E: Risk Of Bias In Non-randomized Studies – of Exposures
T2DM: Type 2 diabetes mellitus
TG/HDL-C: Triglyceride-to-high-density lipoprotein cholesterol ratio
τ^2^: Tau-squared
