# Supplementary material for: Atherogenic lipid indices and diabetic retinopathy in type 2 diabetes: a systematic review and meta-analysis
Source: Front Med (Lausanne). 2026 Jan 9;12:1699408. doi: 10.3389/fmed.2025.1699408 (PMC12827623; doi:10.3389/fmed.2025.1699408)
Supplement: Supplementary file 1 [file Presentation_1.pdf]

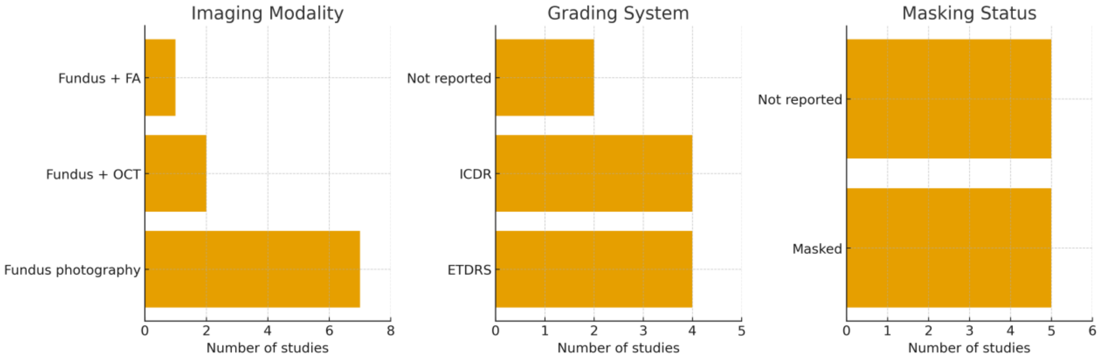

Supplementary Figure S1. Distribution of DR ascertainment methods across included studies. The figure summarizes the number of studies employing each imaging modality (fundus photography, OCT, FA), DR grading system (ETDRS, ICDR, not reported), and masking status (masked vs. not reported). Most studies used fundus photography and standardized ETDRS or ICDR grading, with masking explicitly reported in four of ten studies.

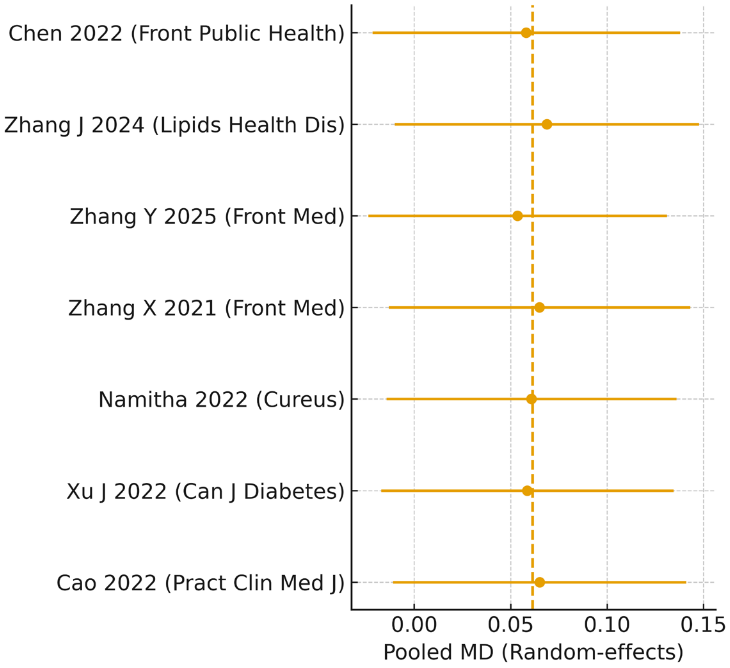

Supplementary Figure 2. Leave-one-out influence analysis for AIP (Random-effects model).
Pooled mean differences (MDs) and 95% CIs are re-estimated after omitting each study in turn under a random-effects model (REML–HKSJ in the main analysis). Results indicate that no single study exerts undue influence on the overall effect.

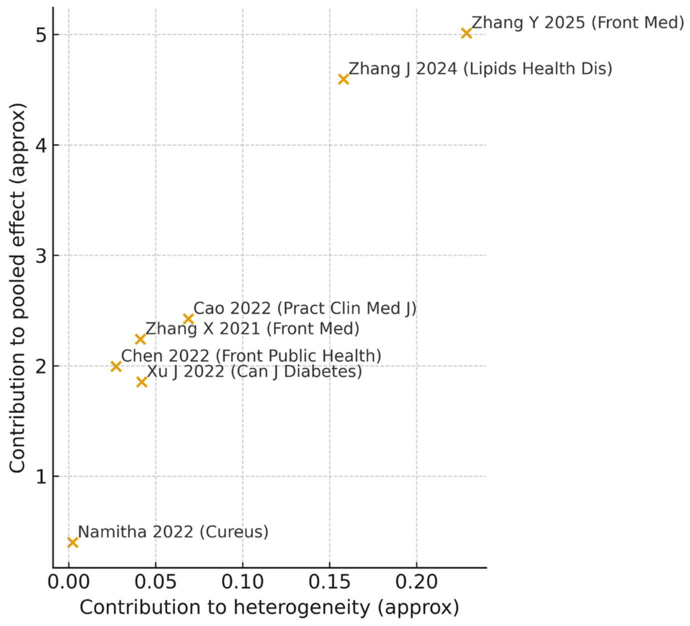

Supplementary Figure 3. Baujat plot for AIP.

Each study's approximate contribution to between-study heterogeneity (x-axis) and to the pooled effect (y-axis) is shown for diagnostic purposes. Studies in the upper-right quadrant contribute more to both heterogeneity and the pooled effect.

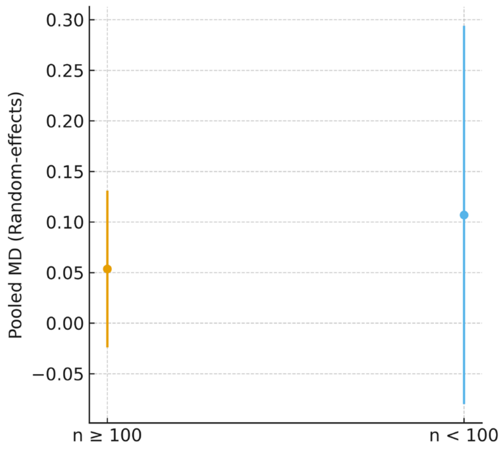

Supplementary Figure 4. Sensitivity analysis stratified by sample size (AIP). Random-effects pooled MDs (95% CIs) are shown for studies with sample size ⩾ 100 vs <100. The consistency across strata supports the robustness of the main findings.

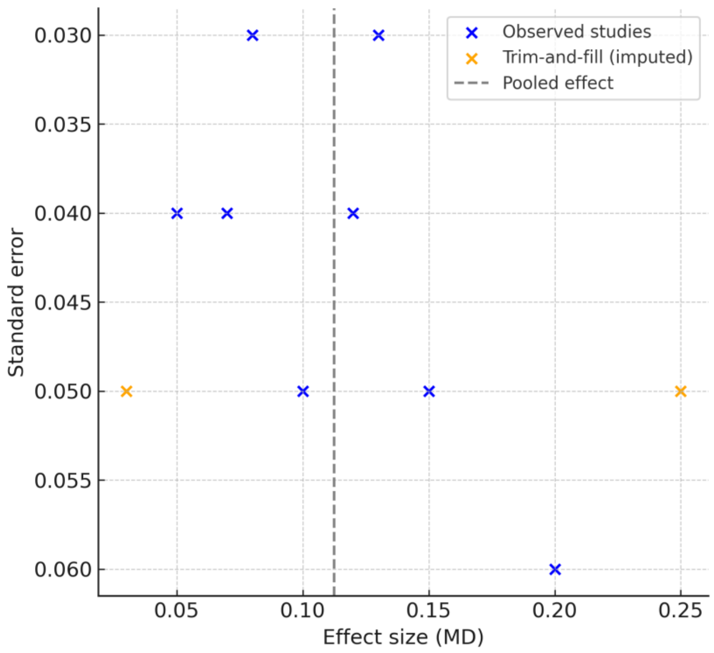

Supplementary Figure S5. Funnel and Trim-and-Fill Plot for AIP Meta-analysis (Exploratory only; <10 studies)
