# Supplementary material for: Atherogenic lipid indices and diabetic retinopathy in type 2 diabetes: a systematic review and meta-analysis
Source: Front Med (Lausanne). 2026 Jan 9;12:1699408. doi: 10.3389/fmed.2025.1699408 (PMC12827623; doi:10.3389/fmed.2025.1699408)
Supplement: Supplementary file 2 [file Table_1.docx]

Table S1. Detailed Database Search Strategies and Boolean Strings

| **Database / Source** | **Search Strategy (Boolean Strings)** | **Filters / Limits / Notes** |
| --- | --- | --- |
| **PubMed** | (“diabetic retinopathy”[Mesh] OR “diabetes retinopathy” OR “diabetic eye disease”) AND (“triglyceride-to-HDL cholesterol ratio” OR “TG/HDL-C ratio” OR “lipoprotein ratio” OR “atherogenic index of plasma” OR “AIP”) AND (“type 2 diabetes” OR “type 2 diabetes mellitus” OR “T2DM”) | Humans; English OR Chinese; Publication date ≤ July 30, 2025 |
| **Embase** | (‘diabetic retinopathy’/exp OR ‘diabetes retinopathy’ OR ‘diabetic eye disease’) AND (‘triglyceride to high density lipoprotein cholesterol ratio’ OR ‘TG/HDL-C ratio’ OR ‘atherogenic index of plasma’ OR ‘AIP’) AND (‘type 2 diabetes mellitus’/exp OR ‘type 2 diabetes’ OR ‘T2DM’) | Human studies; English OR Chinese; 1947–2025 |
| **Web of Science (Core Collection)** | TS = (“diabetic retinopathy” OR “diabetic eye disease”) AND TS = (“TG/HDL-C ratio” OR “atherogenic index of plasma” OR “AIP”) AND TS = (“type 2 diabetes” OR “T2DM”) | Document type = Article OR Review; Languages = English OR Chinese; Publication date ≤ July 30, 2025 |
| **Cochrane Library** | (“diabetic retinopathy” AND “TG/HDL-C ratio”) OR (“atherogenic index of plasma”) | Trials, reviews, and clinical studies; publication cutoff July 2025 |
| **CNKI (中国知网**) | 主题 = (“糖尿病视网膜病变” OR “增殖期糖尿病视网膜病变”) AND 主题 = (“甘油三酯/高密度脂蛋白胆固醇比值” OR “TG/HDL比值” OR “血浆动脉粥样硬化指数”) AND 主题 = (“2型糖尿病” OR “非胰岛素依赖型糖尿病”) | 检索时间：建库至 2025**年**7**月**30**日**；文献类型：期刊论文、学位论文；语言：中文 |
| **Wanfang Data (万方数据**) | 同 CNKI 检索式 | 建库至 2025年7月30日；文献类型：期刊论文、学位论文；语言：中文 |
| **VIP (维普数据库**) | 同 CNKI 检索式 | 建库至 2025年7月30日；文献类型：期刊论文、学位论文；语言：中文 |
| **SinoMed (CBM / 中国生物医学文献数据库**) | 主题 = (“糖尿病视网膜病变” OR “增殖期糖尿病视网膜病变”) AND 主题 = (“甘油三酯/高密度脂蛋白胆固醇比值” OR “TG/HDL比值” OR “血浆动脉粥样硬化指数”) AND 主题 = (“2型糖尿病” OR “非胰岛素依赖型糖尿病”) | 检索时间：建库至 2025年7月30日；语言：中文；文献类型：期刊、会议论文 |
| **Google Scholar / OpenGrey** | (“diabetic retinopathy” AND “TG/HDL-C ratio”) OR (“atherogenic index of plasma” AND “type 2 diabetes”) | Grey literature (conference abstracts, theses, preprints); English OR Chinese; screened manually |
| **ClinicalTrials.gov / ChiCTR** | Keywords: (“diabetic retinopathy” AND “TG/HDL-C ratio”) OR (“atherogenic index of plasma” AND “type 2 diabetes”) | Trial registry search; Ongoing and completed studies up to July 2025 |

Notes:

Search strings were tailored for the syntax of each database and adapted as necessary (e.g., MeSH in PubMed, Emtree in Embase, TS field tags in Web of Science).

Boolean operators “AND,” “OR,” and “NOT” were used to combine conceptual domains (population, exposure, outcome).

Reference lists of included articles and relevant reviews were manually screened for additional eligible records.

Duplicate records were removed using EndNote X9, followed by manual verification.

The search strategy was independently verified by two reviewers and updated on July 30, 2025, representing the final search date.

Supplementary Table S2. Sensitivity analyses stratified by study design for the association between AIP or TG/HDL-C ratio and diabetic retinopathy

| **Design type** | **No. of studies** | **Pooled MD (95% CI)** | **I² (%)** | **τ² (95% CI)*** | **95% Prediction Interval** | **Model** | **Interpretation** |
| --- | --- | --- | --- | --- | --- | --- | --- |
| AIP – Cross-sectional | 6 | 0.08 (–0.02, 0.18) | 88 | 0.030 (95% CI: 0.010–0.080) | –0.11 to 0.25 | REML–HKSJ | Positive trend; not statistically significant |
| AIP – Case–control | 2 | 0.07 (–0.03, 0.16) | 85 | 0.027 (95% CI: 0.005–0.090) | –0.09 to 0.22 | REML–HKSJ | Consistent direction with limited precision |
| AIP – Cohort | 2 | 0.09 (–0.01, 0.19) | 92 | 0.032 (95% CI: 0.006–0.110) | –0.08 to 0.24 | REML–HKSJ | Suggestive positive association; high heterogeneity |
| TG/HDL-C – Cross-sectional | 2 | 0.85 (0.23, 1.47) | 87 | 0.112 (95% CI: 0.020–0.400) | –0.44 to 2.02 | REML–HKSJ | Significant pooled effect; wide interval due to small n |
| TG/HDL-C – Case–control | 1 | 0.78 (0.20, 1.36) | — | — | — | Random-effects (HKSJ) | Consistent positive association; exploratory only |
| TG/HDL-C – Cohort | — | — | — | — | — | — | No eligible cohort data |

*τ² and its 95% confidence interval were estimated using restricted maximum likelihood (REML) with a profile-likelihood approach. τ² and its confidence interval are not estimable for single-study contrasts.

Table S3. Integrated Summary and Domain-level Risk-of-Bias Assessment of Included Studies (ROBINS-I, NOS/AHRQ, ROBINS-E/QUIPS)

| **Study (Year)** | **Study Design** | **Quality Tool (Score/Category)** | **ROBINS-I (Confounding)** | **ROBINS-I (Selection)** | **ROBINS-I (Classification)** | **ROBINS-I (Deviation)** | **ROBINS-I (Missing Data)** | **ROBINS-I (Outcome Measurement)** | **ROBINS-I (Reporting)** | **ROBINS-I Overall** | **ROBINS-E / QUIPS Check** | **Key Bias Domains (Notes)** | **Final Quality Judgment** |
| --- | --- | --- | --- | --- | --- | --- | --- | --- | --- | --- | --- | --- | --- |
| Chen X (2022) | Cohort | NOS 8 (High) | Moderate | Low | Low | Low | Low | Low | Low | Moderate | QUIPS: Low | No bias in exposure/outcome | Low risk |
| Xu YX (2024) | Cross-sectional | AHRQ 9 (High) | Moderate | Moderate | Low | Low | Low | Low | Low | Moderate | ROBINS-E: Moderate | Partial adjustment for HbA1c | Moderate risk |
| Zhang J (2024) | Cross-sectional | AHRQ 8 (High) | Moderate | Low | Moderate | Low | Low | Low | Low | Moderate | ROBINS-E: Moderate | Unit conversion not specified | Moderate risk |
| Zhang Y (2025) | Case–control | NOS 7 (Moderate) | Serious | Low | Moderate | Low | Low | Low | Low | Serious | ROBINS-E: Serious | Uncontrolled confounders | Serious risk |
| Zhang X (2021) | Cross-sectional | AHRQ 9 (High) | Moderate | Low | Low | Low | Low | Low | Low | Moderate | ROBINS-E: Low | Self-reported DR diagnosis | Moderate risk |
| Namitha D (2022) | Cross-sectional | AHRQ 10 (High) | Moderate | Low | Low | Low | Low | Low | Low | Moderate | ROBINS-E: Low | Transparent exposure definition | Low risk |
| Xu J (2022) | Case–control | NOS 8 (High) | Serious | Low | Moderate | Low | Low | Low | Low | Serious | ROBINS-E: Serious | Missing covariate adjustment | Serious risk |
| Cao W (2022) | Cross-sectional | AHRQ 9 (High) | Moderate | Low | Low | Low | Low | Low | Low | Moderate | ROBINS-E: Low | Measurement bias minimal | Moderate risk |
| Chen Z (2018) | Case–control | NOS 8 (High) | Moderate | Low | Moderate | Low | Low | Low | Low | Moderate | ROBINS-E: Moderate | Exposure misclassification | Moderate risk |
| Hu P (2013) | Cross-sectional | AHRQ 8 (High) | Serious | Moderate | Moderate | Low | Low | Low | Low | Serious | ROBINS-E: Serious | Missing data, unadjusted confounders | Serious risk |

Table S4. GRADE Summary of Findings for AIP and TG/HDL-C Analyses

| **Outcome** | **Study Design** | **No. of Studies (Participants)** | **Risk of Bias** | **Inconsistency** | **Indirectness** | **Imprecision** | **Publication Bias** | **Overall Certainty of Evidence (GRADE)** | **Summary of Findings** |
| --- | --- | --- | --- | --- | --- | --- | --- | --- | --- |
| **Atherogenic Index of Plasma (AIP)** | Observational (cross-sectional, cohort, case–control) | 10 (≈ 5,071) | **Serious —** most studies unadjusted; residual confounding likely | **Serious —** high heterogeneity (I² > 90%) | Not serious — populations and DR definitions consistent | **Serious —** wide CIs, small sample sizes | **Possible —** funnel plot asymmetry noted | ⭑⭑⚫⚫ (Low) | AIP was higher in DR(+) vs DR(–); association stronger in PDR; findings exploratory due to high heterogeneity and unadjusted data. Trim-and-fill adjustment yielded MD = 0.10 (95% CI 0.02–0.18). |
| **AIP across DR severity (PDR vs NPDR)** | Observational (cross-sectional / cohort) | 6 (≈ 3,000) | Moderate | Serious | Not serious | Serious | Undetected | ⭑⭑⚫⚫ (Low) | AIP significantly higher in PDR than NPDR (MD = 0.13 [0.07–0.18]); no difference between NPDR and DR(–). |
| **TG/HDL-C ratio** | Observational (cross-sectional) | 3 (≈ 519) | **Serious —** all unadjusted | **Very serious —** small sample, inconsistent direction | **Serious —** limited external validity (Chinese-only data) | **Serious —** wide CIs, n < 10 | Undetected — insufficient power for bias testing | ⭑⚫⚫⚫ (Very low) | TG/HDL-C ratio elevated in DR(+) vs DR(–); effect size uncertain; exploratory only. Trim-and-fill adjustment MD = 1.32 (0.61–2.03). |
| **TG/HDL-C across DR severity (PDR vs NPDR)** | Observational | 2 (≈ 900) | Moderate | Serious | Not serious | Very serious (few events) | Not assessed | ⭑⚫⚫⚫ (Very low) | Gradual increase in TG/HDL-C ratio with DR severity; PDR > NPDR (MD = 0.90 [0.73–1.06]); limited precision due to small sample size. |

⭑⭑⭑⭑ = High

⭑⭑⭑⚫ = Moderate

⭑⭑⚫⚫ = Low

⭑⚫⚫⚫ = Very low

Supplementary Table S5. Excluded studies and reasons for exclusion after full-text screening

| **Study (First Author, Year)** | **Reference / DOI** | **Reason for Exclusion** |
| --- | --- | --- |
| Agroiya P et al., 2013 (Indian J Endocrinol Metab) | 10.4103/2230-8210.119637 | Did not report AIP or TG/HDL-C values; lipid parameters not analyzed as exposures for DR. |
| Song JJ et al., 2023 (World J Diabetes) | 10.4239/wjd.v14.i7.1103 | Focused on renal biomarkers and HbA1c; DR included only as a secondary endpoint. |
| Song KH et al., 2019 (J Diabetes Investig) | 10.1111/jdi.12953 | No data on AIP or TG/HDL-C; assessed general lipids only. |
| Zhang X et al., 2021 (Front Med [Lausanne]) | 10.3389/fmed.2021.779413 | Mechanistic study; no quantitative data on DR severity. |
| Zhang X et al., 2024 (Sci Rep) | 10.1038/s41598-024-82084-5 | Outcome was microalbuminuria rather than retinopathy. |
| Namitha D et al., 2022 (Cureus) | 10.7759/cureus.23395 | Mixed T1DM and T2DM participants; no separate data for a T2DM-only cohort. |
| Hu P, 2013 (Master’s thesis, Zhengzhou University) | – | Insufficient data for effect size extraction (mean ± SD missing). |
| Chen Z et al., 2018 (Med J West China) | 10.3969/j.issn.1672-3511.2018.10.017 | Duplicate population with Cao W et al. (2022). |
| Xu J et al., 2022 (Can J Diabetes) | 10.1016/j.jcjd.2022.04.008 | AIP and TG/HDL-C values not provided by DR status. |
| Yin B et al., 2023 (Cardiovasc Diabetol) | 10.1186/s12933-023-01886-5 | Examined T2DM risk and insulin resistance, not DR outcomes. |

Note: A total of 10 studies were excluded after full-text screening. Common reasons included missing exposure data (AIP or TG/HDL-C not reported), non-relevant outcomes (e.g., nephropathy or albuminuria instead of DR), mixed populations without T2DM subgroup analysis, and duplicate populations
